# Involvement of RbAp48 in erythroid differentiation of murine erythroleukemia cells induced by sodium butyrate

**DOI:** 10.3892/ol.2014.2015

**Published:** 2014-03-31

**Authors:** WU-LIN QI, LING-LING CAO, JIANG-JIANG HU, JIAN-YOU XUE, TING-TING SANG, YA-JUAN ZHENG, TAO CHEN, JIE WANG, FU-KUN ZHAO, SHI-FU ZHANG

**Affiliations:** College of Life Sciences, Zhejiang Sci-Tech University, Hangzhou, Zhejiang 310018, P.R. China

**Keywords:** RbAp48, murine erythroleukemia cell, sodium butyrate, erythroid differentiation

## Abstract

Normal mammalian terminal erythroid differentiation is a precisely regulated process during which the progenitor cells execute particular programs to form a mature erythrocytic phenotype. In the present study, it was found that RbAp48, a histone-binding protein associated with retinoblastoma protein, was upregulated during terminal erythroid maturation *in vivo* and *in vitro*. This indicated that RbAp48, at least in part, participated in the regulation of murine erythropoiesis. Following sodium butyrate (SB) induction, murine erythroleukemia (MEL) cells began to re-enter erythroid differentiation and the ratio of differentiated cells reached ~80% at 72 h. The erythroid maturation-related mRNA expression of α-globin, β-globin and glycophorin A (GPA) was increased markedly, which indicated that SB induced MEL differentiation. During MEL differentiation, the RbAp48 level showed a 1.5-fold increase at 72 h, and the globin transcription factor (GATA)-1 level was also upregulated in the early stage of differentiation. By contrast, the c-Myc level was gradually downregulated in MEL differentiation. Using an immunofluorescence assay, the results of the study directly showed that the average fluorescence intensity of RbAp48 in each cell reached an almost 1.7-fold increase at 72 and 96 h. This was consistent with the western blot results of RbAp48 during MEL differentiation. In addition, reduced expression of RbAp48 by RNA inference decreased SB-induced MEL differentiation by ~20%, indicating that a high level of RbAp48 was essential for MEL differentiation. Taken together, these results established a functional link between RbAp48 and erythroid differentiation.

## Introduction

RbAp48, a member of the WD-40 protein family that is characterized by its ability to bind to the retinoblastoma protein (Rb), was first identified from the HeLa cell lysate (1). RbAp48 is involved in the regulation of cytoskeletal organization. The overexpression of RbAp48 in breast cancer cells results in profound changes of cellular morphology, including cell size reduction, decreased cellular protrusions and a circular cell shape (2). In addition, small interfering RNA-mediated depletion of RbAp48 in HPV16-immortalized human cervical mucosa epithelial H8 cells dramatically stimulates cell growth and colony formation (3). Previously, numerous studies have demonstrated that the level of RbAp48 is changed in liver cancer (4), thyroid carcinoma (5) and acute myeloid leukemia (6) cells when compared with normal cells. These studies indicated that RbAp48 may play significant roles in cell cycle and tumor formation. RbAp48, together with the hematopoietic transcription factor, GATA-1, and several other proteins functions early to repress the genes required to maintain G1E cells in the undifferentiated state, which contributes to terminal erythroid differentiation (7). Additionally, GATA-1 is able to mediate the transcription of erythroid-specific genes and repress the proliferation-related c-Myc gene in G1E-ER4 cells (8). c-Myc plays key roles in normal, non-transformed cells in regulating cell growth, differentiation and apoptosis.

However, little is known about the function of RbAp48 in the regulation of cell differentiation. Our previous experiments discovered that the expression level of RbAp48 in fetal livers was increased during terminal erythroid differentiation through comparative proteomic analysis (data not published). In the present study, the changes in the cellular level and localization of RbAp48 during terminal erythroid maturation were examined. Murine erythroleukemia (MEL) differentiation in culture induced by SB provides an ideal model for studying differentiation-associated proteins. By means of this model, the present study attempted to preliminarily elucidate the function of RbAp48 in MEL differentiation.

## Materials and methods

### Cell and cell culture

HEK293T and MEL cells were purchased from the Type Culture Collection of the Chinese Academy of Science (Shanghai, China) and were cultured in Dulbecco’s modified Eagle’s medium (Gibco; Invitrogen Life Technologies, Carlsbad, CA, USA) supplemented with 10% fetal bovine serum (Hyclone; Thermo Fisher Scientific, Rockford, IL, USA) at 37°C in a humidified atmosphere of 5% CO_2_.

### Benzidine staining

Erythroid differentiation was evaluated by the expression of hemoglobin, which can be detected by benzidine staining. The MEL cells were suspended in a benzidine staining solution (0.4 mg/ml benzidine in 0.6% H_2_O_2_, 3% acetic acid and 8.5 g/l NaCl). Following 2 min of staining, the differentiated cells were stained blue and images were captured by an inverted fluorescence microscope (Nikon, Tokyo, Japan). The percentage of benzidine-positive cells was the number of blue cells divided by the total number of cells.

### MTT assay

To measure cell proliferation activity, each group of MEL cells was seeded in 96-well plates at a density of 3×10^3^ cells per well. The MTT assay was performed at 24, 48, 72, 96 and 120 h. To each well, 10 μl MTT (5 mg/ml) was added and the cells were incubated for an additional 4 h in the cell culture incubator. The medium in each well was then replaced with 150 μl dimethylsulfoxide and the absorbance was measured at 570 nm on a spectrophotometer (Thermo Fisher Scientific).

### Semi-quantitative polymerase chain reaction (PCR)

Total RNA was extracted from each cell sample. The cDNA was synthesized from 0.5 μg total RNA using a reverse transcription kit (Fermentas; Thermo Fisher Scientific). The number of PCR cycles was optimized in each case to guarantee that the product intensity fell within the linear range of amplification. PCR amplification was performed as follows: initial denaturation at 94°C for 5 min, followed by additional denaturation at 94°C for 30 sec, annealing at 50°C for 30 sec, extension at 72°C for 30 sec. At the end of the cycle, the reaction mixtures were maintained at 72°C for a further 3 min. The primers are listed in Table I. The PCR products were analyzed by agarose gel electrophoresis with β-actin as an internal control.

### Western blot analysis

The cells were washed with ice-cold phosphate-buffered saline (PBS) and lysed by lysis buffer (50 mM Tris, 150 mM NaCl, 1 mM EDTA, 1% Triton 100 and 0.1% SDS; pH 8.0) for 20 min on ice. Protein concentrations were determined by Bradford assay. Equal amounts of proteins were subjected to 12% SDS-PAGE and transferred onto polyvinylidene difluoride membranes. The membranes were blocked in Tris-buffered saline and Tween 20 (TBST; 50 mM Tris, 150 mM NaCl and 0.1% Tween-20; pH 7.5) containing 5% skimmed dry milk for 2 h at room temperature, and then incubated with primary anti-RbAp48 rabbit monoclonal (Epitomics, Inc., Burlingame, CA, USA), anti-c-Myc (Epitomics, Inc.), anti-GATA-1 rabbit monoclonal (CST, Boston, MA, USA) or anti-β-actin rabbit monoclonal (Sigma-Aldrich, St. Louis, MO, USA) antibodies overnight at 4°C. The membranes were then washed and incubated with goat anti-rabbit immunoglobulin G (IgG)-horseradish peroxidase antibody (Sigma-Aldrich) for 2 h at room temperature. The proteins were visualized by chemiluminescence using an enhanced chemiluminescence kit (Advansta, Inc., Menlo Park, CA, USA). Immunoblots were quantified by Quantity One software (Bio-Rad, Hercules, CA, USA).

### Immunofluorescence assay

The MEL cells were harvested at 0, 24, 48, 72, and 96 h following treatment with 1.25 mM SB. Subsequent to being fixed in 4% paraformaldehyde for 15 min, the cells were permeabilized with methanol for 10 min, stained with DAPI for 15 min at 37°C and blocked in 5% bovine serum albumin for 30 min at room temperature. The cells were incubated with anti-RbAp48 antibody overnight at 4°C, washed with PBS Tween-20 and then incubated with cyanine dye 3-conjugated goat anti-rabbit IgG antibody (Invitrogen Life Technologies) for 2 h at room temperature. The images of stained cells were captured using confocal fluorescence microscopy (Nikon).

### Generation of the stable RbAp48-knockdown cell line

For knockdown of the RbAp48 gene by RNA interference, the following oligonucleotide pair was designed: Small hairpin (sh)RNA sense, 5′-CCG GCC CTG CAT CAT TGC AAC AAA GCT CGA GCT TTG TTG CAA TGA TGC AGG GTT TTT G-3′ and antisense, 5′-AAT TCA AAA ACC CTG CAT CAT TGC AAC AAA GCT CGA GCT TTG TTG CAA TGA TGC AGG G-3′. As a control, a scramble sequence of shRNA was also designed: shRNA-negative control sense, 5′-CCG GCC TAA GGT TAA GTC GCC CTC GCT CGA GCG AGG GCG ACT TAA CCT TAG GTT TTT G-3′ and antisense, 5′-AAT TCA AAA ACC TAA GGT TAA GTC GCC CTC GCT CGA GCG AGG GCG ACT TAA CCT TAG G-3′. Two double-stranded oligonucleotides were inserted into the plasmid vector, pLKO.1-TRC (Addgene, Cambridge, MA, USA), via *Age*I and *Eco*RI restriction sites. All different lentiviruses were produced by co-transfection of 293T cells with pLKO.1-RbAp48-shRNA or pLKO.1-shRNA-NC vectors and the ecotropic packaging vectors, pCMV-VSVG and pCMV-dR8.2, using Lipofectamine 2000 (Invitrogen Life Technologies), and then the lentiviruses were used to transfect the MEL cells. Stable RbAp48-knockdown cells were selected in 1 μg/ml puromycin (Invitrogen Life Technologies).

### Statistical analysis

SPSS Statistics 19 software (IBM, Armonk, NY, USA) was used for statistical analysis. Data were presented as the mean ± standard deviation for three different determinations. Statistical significance was analyzed using the one-way analysis of variance test followed by Fisher’s least significant difference test. P<0.05 was considered to indicate a statistically significant difference.

## Results

### Erythroid differentiation of MEL cells induced by SB

**Figure 1:**
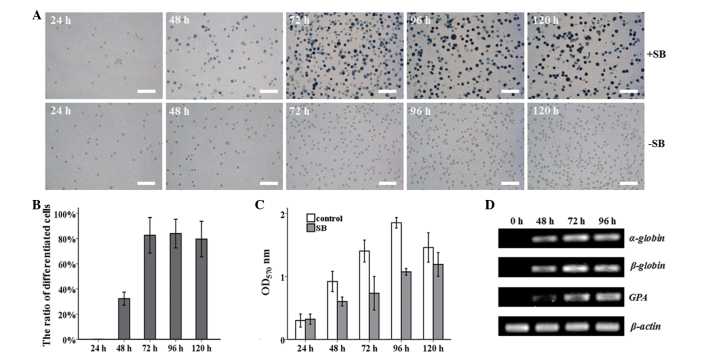
SB-induced erythroid differentiation of MEL cells. (A) MEL cells were harvested after 24, 48, 72, 96, and 120 h with or without 1.25 mM SB induction, and stained with benzidine. Benzidine-positive cells turned blue. Untreated MEL cells served as a control. White bar, 100 μm. (B) The percentage of benzidine-positive cells was counted at different times. ^*^P<0.001 vs. control group. Control group, MEL cells without SB treatment at various time points. (C) The effect of 1.25 mM SB on the MEL cell proliferation ability was measured by MTT assay. ^*^P<0.05 and ^**^P<0.01 vs. control group. Control group, MEL cells without SB treatment at various time points. (D) The mRNA expression of α-globin, β-globin and GPA was dramatically increased during MEL differentiation, as assayed by semi-quantitative PCR. β-actin was used as an internal control. Three independent experiments were performed. SB, sodium butyrate; MEL, murine erythroleukemia; OD, optical density; PCR, polymerase chain reaction; GPA, glycophorin A.

Expression of hemoglobin was the most evident feature of erythroid differentiation. A preliminary study showed that the optimum concentration of the inducer, SB, was 1.25 mM, which was used in subsequent experiments (data now shown). Benzidine staining was used to detect the expression of hemoglobin in MEL cells (Fig. 1A). Almost 80% of the cells had differentiated following treatment with SB for 72 h (Fig. 1B). However, no benzidine-positive cells were observed in the untreated cells. The MEL cells, in the presence of SB, could initiate erythroid differentiation at the expense of proliferation (Fig. 1C). During MEL differentiation, the erythroid maturation-related mRNA expression of α-globin, β-globin and GPA was increased markedly, and reached a plateau at 72 h (Fig. 1D).

### An elevation of RbAp48 level and the changes of GATA-1 and c-Myc level during terminal erythroid maturation

**Figure 2:**
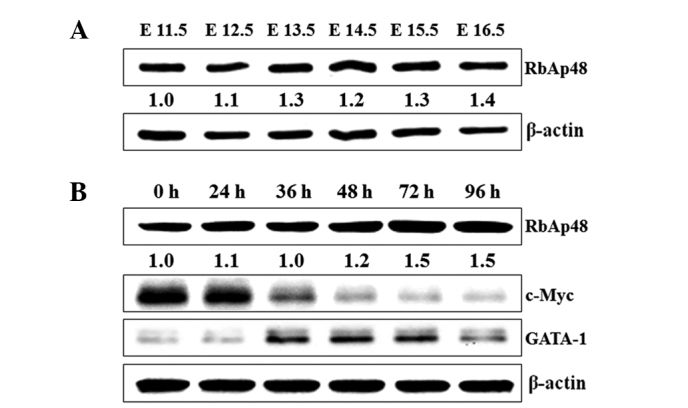
Detection of RbAp48, GATA-1 and c-Myc expression in erythroid differentiation. (A) The RbAp48 level during terminal erythroid differentiation in fetal liver from E11.5 to E16.5 was assessed by western blot analysis. (B) Total proteins extracted from MEL cells with 1.25 mM SB induction at 0, 24, 36, 48, 72 and 96 h were used to detect RbAp48, GATA-1 and c-Myc levels by western blot analysis. β-actin was used as an internal control. Three independent experiments were performed. SB, sodium butyrate; MEL, murine erythroleukemia.

Since our previous experiments had indicated that the level of RbAp48 in the fetal liver was changed during murine embryonic development, we hypothesized that RbAp48 may function in terminal erythroid maturation. To test this prediction, fetal livers were isolated from imprinting control region (ICR) mouse embryos of E11.5–16.5 stage, and the RbAp48 expression level was measured. A gradual increase in RbAp48 was detected in the fetal liver from E11.5 to E16.5 (Fig. 2A). Furthermore, it was found that the RbAp48 expression also increased in MEL cells induced by SB, and GATA-1 and c-Myc levels were changed significantly. The GATA-1 level showed a rapid increase in the early stage of differentiation, and the c-Myc level was gradually downregulated during MEL differentiation (Fig. 2B). Therefore, RbAp48, GATA-1 and c-Myc may play significant roles in erythroid differentiation.

### Cellular location of RbAp48 during erythroid differentiation

**Figure 3:**
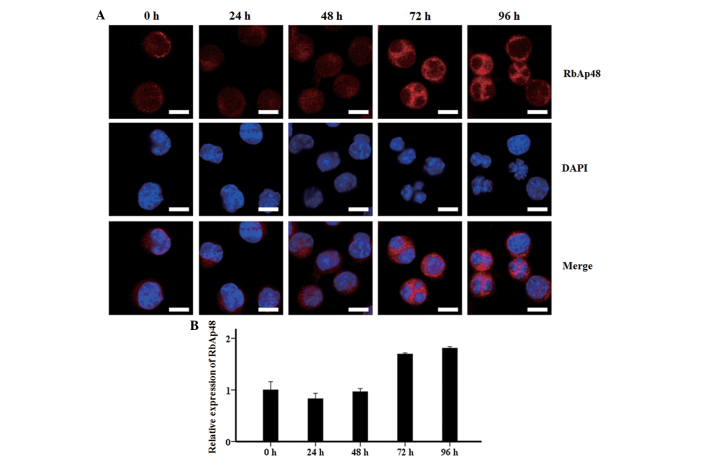
Cellular localization of RbAp48 during MEL differentiation. (A) MEL cells with 1.25 mM SB induction were harvested at 0, 24, 48, 72 and 96 h and stained with anti-RbAp48 (red) antibody and DAPI (blue) by immunofluorescence assay using confocal microscopy. White bar, 10 μm. (B) Quantitative analysis with EZ-C1 3.20 software detected the fluorescence intensity of RbAp48 in each MEL cell. Three independent experiments were performed. ^*^P<0.05 vs. control group. Control group, MEL cells without SB treatment. MEL, murine erythroleukemia; SB, sodium butyrate.

Confocal microscopic observations showed that the RbAp48 protein was mainly distributed in the nucleus within 48 h of SB treatment, and rapidly accumulated in the cytoplasm from 72 to 96 h (Fig. 3A). Quantitative analysis with EZ-C1 3.20 software illustrated that the fluorescence intensity of RbAp48 in each cell remained stable and at a low level from 0 to 48 h, prior to an increase to 1.7 fold at 72 and 96 h (Fig. 3B). This was consistent with the western blot analysis results of RbAp48.

### Suppression of RbAp48 expression results in significant prevention of MEL cell differentiation induced by SB

**Figure 4:**
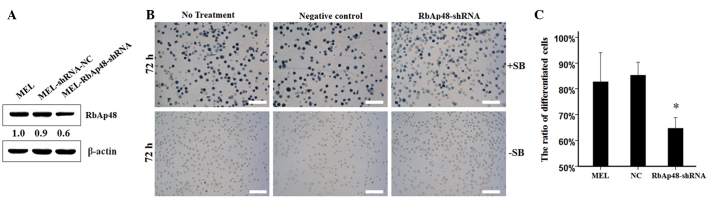
Reduced expression of RbAp48 inhibits MEL differentiation. (A) Proteins extracted from MEL, MEL-shRNA-NC and MEL-RbAp48-shRNA were used to detect the RbAp48 level by western blot analysis. (B) Benzidine staining of untransfected and transfected MEL cells was shown following 72 h with or without 1.25 mM SB induction. Bar, 100 μm. (C) The fraction of benzidine-positive cells was determined by a benzidine cytochemical test. Data were obtained from three independent experiments. ^*^P<0.05 vs. vs. control or negative control. Control, parent MEL cells; negative control, MEL cells transfected with control shRNA. MEL, murine erythroleukemia; SB, sodium butyrate, shRNA, small hairpin RNA; NC, negative control.

To determine whether a specific level of RbAp48 was required for the erythroid differentiation of MEL cells, the expression of the RbAp48 gene in MEL cells was suppressed using the RNA interference (RNAi) approach. The stable RbAp48-knockdown cell line was verified by western blot analysis. MEL-RbAp48-shRNA resulted in a 0.6-fold reduction of endogenous RbAp48 when compared with MEL-shRNA-NC and parent MEL cells (Fig. 4A). To further investigate the effect of the knockdown of RbAp48 on cell differentiation, benzidine staining was performed on the cultured cells incubated with SB for 72 h, and there were no benzidine-positive cells in the absence of SB (Fig. 4B). The results of the present study revealed that the knockdown of RbAp48 expression in the MEL cells decreased the differentiation ability by ~20% (Fig. 4C).

## Discussion

Leukemia, a malignant hematopoietic system disease, is mainly the result of a disorder of the hematopoietic stem cells in differentiation and apoptosis (9). However, studies have indicated that SB, a histone deacetylase inhibitor, exhibits anticancer effects via the apoptosis and differentiation of cancer cells (10). In the present study, MEL cells, when cultured in the presence of SB, chose the differentiation pathway and synthesized erythroid markers, including α-globin, β-globin and GPA. The effect of SB on the proliferation characteristic of the MEL cells was also detected. SB evidently suppressed the cell proliferation ability. Therefore, SB induced MEL differentiation at the expense of proliferation, accompanied by the expression of erythroid markers.

RbAp48 was found to be upregulated in the fetal liver from E11.5 to E16.5. Furthermore, it also showed a steady increase upon SB induction in the MEL cells. This specific expression in certain phases indicated that RbAp48 was involved in cell differentiation. During MEL differentiation, GATA-1 and c-Myc levels were also changed significantly. The GATA-1 level showed a rapid increase in the early stage of differentiation, and the c-Myc level was gradually downregulated during MEL differentiation. The GATA-1/RbAp48 complex has been proven to promote erythroid differentiation in G1E cells (7). GATA-1-mediated c-Myc transcriptional repression is due to the direct interaction in the c-Myc promoter (8). Repression of c-Myc has been linked to proliferation cessation, and evidence has demonstrated that the downregulation of c-Myc is essential for terminal erythroid maturation (11). Therefore, RbAp48, GATA-1 and c-Myc may play significant roles in erythroid differentiation.

Next, the present study investigated the cellular localization of RbAp48 during erythroid differentiation. Prolonging the SB induction time rapidly increased the content of RbAp48 in the cytoplasm at 72 and 96 h. This indicated that RbAp48 showed marked expression in the late stage of erythroid differentiation. In short, through western blot analysis and immunofluorescence assays, it was found that the RbAp48 level was upregulated during MEL differentiation. Therefore, a high level of RbAp48 may contribute to MEL differentiation. To further study the effect of low level RbAp48 on MEL differentiation, a stable RbAp48-knockdown cell line was isolated from the MEL cells by the RNAi method. The results of the present study revealed that a low level of RbAp48 blocked the erythroid differentiation of the MEL cells. This result further indicated that a high level of RbAp48 was essential for erythroid differentiation, and that RbAp48 may be a significant differentiation factor.

In the present study, MEL cells could re-enter the erythroid program and obtain the capability to synthesize hemoglobin and GPA in the presence of SB. The expression level of RbAp48 was found to be upregulated during terminal erythroid differentiation, and a relatively low expression level of RbAp48 in MEL cells partly contributed to erythroid differentiation cessation. This indicates the novel role of RbAp48 in regulating MEL differentiation. Advances in the research of RbAp48 in erythroid differentiation will extend our understanding of the mechanisms of SB-induced MEL differentiation.

## Figures and Tables

**Table I:** Sequence of PCR primers

Gene	Primer sequence
α-globin	F: AAGCAACATCAAGGCTGCCT R: ACCTTCTTGCCGTGACCCTT
β-globin	F: AACTCTGGGAAGGCTCCTGA R: TGCAGCTCACTGAGATGAGC
GPA	F: GCTGCTGTGACAACATCAGG R: CAGTAGGGGCCGTGTGATAA
β-actin	F: GAGACCTTCAACACCCCAGC R: ATGTCACGCACGATTTCCC

GPA, glycophorin A; F, forward; R, reverse.
